# Supplementary material for: Repeatome Analyses and Satellite DNA Chromosome Patterns in Deschampsia sukatschewii, D. cespitosa, and D. antarctica (Poaceae)
Source: Genes (Basel). 2022 Apr 26;13(5):762. doi: 10.3390/genes13050762 (PMC9141916; doi:10.3390/genes13050762)
Supplement: Supplementary file 1 [file genes-13-00762-s001.zip › genes-1676201-supplementary.pdf]

Table S1. FISH chromosome mapping of the *D. sukatschewii* satDNA probes in the studied *Deschampsia* species

SatDNA Probe	*D. sukatschewii*	*D. cespitosa*	*D. antarctica*
Ds 52	Multiple clustered and dispersed hybridization signals localized in different chromosome regions, partially overlapping with Ds 81 and Ds 56 signals	Multiple clustered and dispersed signals localized in different chromosome regions, overlapping with Ds 81 and Ds 56 signals	Four clustered signals localized on chromosome pairs 5 (distal region of the satellite) and 12 (distal region of the long arm), overlapping with Ds 81 and Ds 56 signals
Ds 56	Multiple clustered and dispersed signals localized in different chromosome regions, partially overlapping with Ds 81 and Ds 52 signals	Multiple clustered and dispersed signals localized in different chromosome regions, overlapping with Ds 81 and Ds 52 signals	Four clustered signals localized on chromosome pairs 5 (distal region of the satellite) and 12 (distal region of the long arm), overlapping with Ds 81 and Ds 52 signals
Ds 65	Middle-sized clustered signals localized in terminal regions of chromosome pairs 3, 10 and 13, and also in pericentromeric regions of chromosome pairs 1, 2 and 7	Middle-sized clustered signals localized in terminal regions of chromosome pairs 3, 10, 11, 12 and 13, and also in pericentromeric regions of chromosome pairs 2 and 7	Middle-sized clustered signals localized in terminal regions of chromosome pairs 1, 7, 8, 11 and 13, and also in pericentromeric regions of chromosome pairs 2, 3 and 6
Ds 81	Multiple clustered and dispersed hybridization signals localized in different chromosome regions, partially overlapping with Ds 52 and Ds 56 signals	Multiple clustered and dispersed hybridization signals localized in different chromosome regions, overlapping with Ds 52 and Ds 56 signals	Four clustered hybridization signals localized on chromosome pairs 5 (distal region of the satellite) and 12 (distal region of the long arm), overlapping with Ds 52 and Ds 56 signals
Ds 83	Multiple clustered signals localized in the distal regions of most chromosomes	Multiple clustered signals localized in the distal regions of most chromosomes	Multiple clustered signals localized in the distal regions of most chromosomes
Ds 88	Multiple very small clusters and dispersed signals localized along most chromosomes	Middle-sized clustered signals localized in the terminal regions (seven chromosome pairs) and also in pericentromeric regions of one large chromosome pair	Small and middle-sized clustered signals localized in different chromosome positions of eight chromosome pairs
Ds 124	Multiple clustered and dispersed hybridization signals localized in different chromosome regions	Multiple clustered and dispersed hybridization signals localized in different chromosome regions	Clustered signals localized in the proximal regions of the long arms of chromosome pair 11
Ds 138	Very small clustered and dispersed signals localized along most chromosomes	Small and middle-sized clustered signals localized in different chromosome position of seven chromosomes pairs	Multiple small and middle-sized clusters localized in different chromosome positions and also dispersed signals detected along most chromosomes
Ds 144	Multiple small and middle-sized clustered signals localized in different chromosome positions and	Multiple small clustered and dispersed signals localized along most chromosomes	Multiple small and middle-sized clustered signals localized in different chromosome positions

	also dispersed signals detected along most chromosomes		
Ds 146	Middle-sized clustered signals localized in different chromosome positions of chromosome pairs 1, 2, 4, 7, 8, 11 and 13	Middle-sized and small clustered signals localized in different chromosome positions of chromosome pairs 1, 2, 3, 4, 7, 8, 9, 11 and 13	Large and middle-sized clustered signals localized in different chromosome positions of chromosome pairs 1, 2, 3, 4, 6, 7, 11, 12 and 13
Ds 179	Multiple very small clusters and dispersed signals localized along most chromosomes	Small and middle-sized clustered signals localized on seven chromosome pairs in different chromosome positions	Middle-sized clustered signals localized in terminal regions of four chromosome pairs
Ds 226	Middle-sized clusters localized on eight chromosome pairs in different positions and dispersed signals along chromosomes	Multiple small clusters and dispersed signals localized along most chromosomes	Large and middle-sized clustered signals localized on nine chromosome pairs in different chromosome positions
